# Satisfaction with follow-up consultations among Swiss childhood cancer survivors: A prospective cohort study

**DOI:** 10.1016/j.pmedr.2026.103591

**Published:** 2026-07-27

**Authors:** Sara Buchmüller, Sonja Kälin, Martina Ospelt, Gisela Michel, Carole E. Aubert, Jörg D. Leuppi, Maria M. Wertli, Eva Maria E. Tinner

**Affiliations:** a Department of General Internal Medicine, Inselspital, Bern University Hospital, University of Bern, Bern, Switzerland; b Faculty of Health Sciences and Medicine, University of Lucerne, Lucerne, Switzerland; c University Institute of Internal Medicine, Cantonal Hospital Baselland, Liestal, Switzerland; and Medical Faculty University of Basel, Basel, Switzerland; d Institute for Primary Healthcare (BIHAM), University of Bern, Bern, Switzerland; e Department of Internal Medicine, Cantonal Hospital Baden, Baden, Switzerland; f Division of Hematology/Oncology, Department of Pediatrics, Inselspital Bern, University of Bern, Bern, Switzerland

**Keywords:** Childhood cancer survivor, Long-term follow-up, Patient-reported outcomes, Adherence to follow-up, Satisfaction with care, Worries

## Abstract

**Objective:**

Late effects are frequent among adult childhood cancer survivors (ACCS), making long-term follow-up (LTFU) care essential. This study examined satisfaction and worries among ACCS after their first LTFU visit and explored associated factors.

**Methods:**

ACCS (≥18 years) entering LTFU care at two Swiss outpatient clinics, Cantonal Hospital of Baselland and Bern University Hospital, completed questionnaires before and three months after their first visit. Data were collected between 2017 and 2023. Outcomes included satisfaction with patient-centered care and information dissemination, as well as worries, each rated from 1 to 4. Univariable and multivariable regression analyses were used to identify associated factors.

**Results:**

A total of 145 ACCS were included in the study. Satisfaction was very high, with mean scores of 3.77 (range = 2.75–4.00) for patient-centered care and 3.58 (range = 1.78–4.00) for information dissemination. Worries were reported at moderate levels (M = 2.02, range = 1.00–3.67). Discussing a higher proportion of wished topics during the visit was associated with greater satisfaction with patient-centered care (*p* = 0.01). Lower mental health-related quality of life (HRQoL) was associated with increased worries (*p* = 0.03).

**Conclusions:**

Satisfaction with LTFU visits was generally very high. ACCS with lower mental HRQoL may benefit from additional psychological support to mitigate worries.

## Highlights

• Ongoing follow-up care is crucial for survivors of childhood cancer.
• Satisfaction with the first follow-up visit was very high among survivors.
• Discussing topics important to survivors increased satisfaction with care.
• Some survivors experienced moderate worries after follow-up visits.
• Poorer mental well-being was linked to greater worries after the visit.

## 1 Introduction

Most adult childhood cancer survivors (ACCS) experience late effects resulting from their oncological disease and its treatment (Bhakta et al., 2017), making specialized long-term follow-up (LTFU) care crucial. While survival rates have improved, they often come at a high cost, as late effects manifest in various and sometimes severe forms. Late effects may affect several organ systems, particularly the cardiovascular and endocrine systems, and can also include psychosocial difficulties such as anxiety or post-traumatic stress disorder (Bhakta et al., 2017; Bhatia et al., 2023; Bitsko et al., 2016).

To identify late effects of childhood cancer and provide necessary support, LTFU care for childhood cancer survivors is essential. Individualized LTFU aims not only to reduce mortality and morbidity through early detection of late effects and optimized management of chronic toxicities (Skinner et al., 2007), but also to enhance quality of life (Hendriks et al., 2021). Different guidelines outline the core components of LTFU care (Kremer et al., 2013), with the most comprehensive being the Children's Oncology Group LTFU (COG-LTFU) guidelines (Children's Oncology Group, 2023). The individualized follow-up plan is documented in a survivorship passport, such as the *Passport for care*® (PfC; Poplack et al., 2014).

To improve the health of ACCS and reduce the long-term late effects of cancer and its treatment, it is essential to ensure ACCS' continued participation in LTFU care. Sustained participation may be supported by fostering patient satisfaction, addressing worries, and offering individualized care. Previous studies indicated that factors such as the number of topics discussed during consultations, access to psychosocial support, shorter waiting times, and longer consultations were positively associated with patient satisfaction (Absolom et al., 2006; Michel et al., 2011). Importantly, examining these associations across different national and healthcare contexts allows for the identification of both common and context-specific determinants of satisfaction. Since satisfaction may influence adherence to LTFU care, systematically gathering patient feedback is essential to evaluate whether care is meeting ACCS' needs. These insights help tailor LTFU care to diverse survivor needs and identify those at risk of low satisfaction or greater worries.

This study aimed to (1) assess satisfaction and worries following LTFU consultations among ACCS receiving specialized care at one of two Swiss clinics (Liestal or Bern), and (2) identify sociodemographic, cancer-related, consultation-related, and psychosocial characteristics associated with satisfaction and worries.

## 2 Methods

### 2.1 Study design and population

This study used data from a larger cohort of ACCS receiving LTFU care in Liestal or Bern, previously described elsewhere (Kull et al., 2025; Tinner et al., 2024). In Switzerland, several healthcare centers have recently implemented systematic LTFU care through different models (Tinner et al., 2019). At the Cantonal Hospital of Baselland in Liestal and Bern University Hospital, LTFU programs for ACCS were established in 2017 and 2018 and are based on collaboration between internists and pediatric oncologists. Oncological records are reviewed to create individualized follow-up plans according to COG-LTFU guidelines, documented in the PfC, and organized according to survivor needs (Tinner et al., 2024). All ACCS attending their first consultation at one of the participating adult LTFU clinics were invited to participate in the current study. Eligible participants were ACCS aged ≥18 years, diagnosed with cancer before age 20 according to the International Classification of Childhood Cancer—Third Edition (ICCC-3; Steliarova-Foucher et al., 2005), attending LTFU care in Liestal or Bern, and provided informed consent. Exclusion criteria were age < 18 years at first visit and lack of consent.

Referrals occurred through direct transition from pediatric oncology, physician referrals, or self-registration. This first visit did not necessarily represent a direct transition from pediatric to adult care. Consultations were conducted by experienced internists following pediatric oncology follow-up guidelines. Data were collected between 2017 and 2023 using REDCap (Research Electronic Data Capture; https://projectredcap.org/). The reporting of this study adheres to the Strengthening the Reporting of Observational Studies in Epidemiology checklist for cohort studies (STROBE) guidelines (Von Elm et al., 2007).

### 2.2 Procedure

**Fig. 1:**
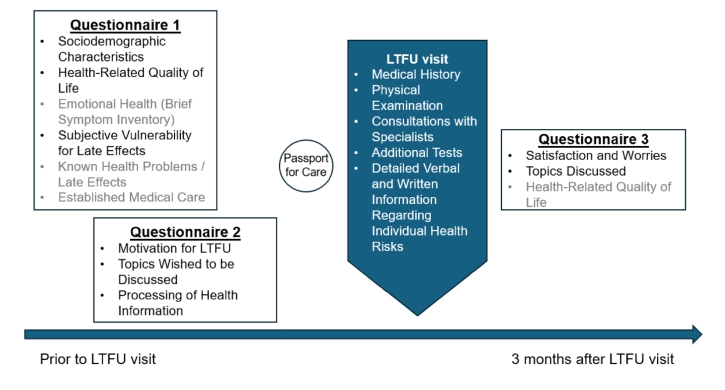
Study procedure and measures in a study of adult childhood cancer survivors attending long-term follow-up clinics in Switzerland, 2017–2023. *Note*. Black elements indicate measures included in the present study; other measures were part of the larger research project. LTFU = long-term follow-up.

Participants completed three questionnaires. Questionnaire 1, administered three months before the LTFU visit, assessed sociodemographic and health characteristics. Questionnaire 2, administered two to four weeks before the visit, assessed LTFU expectations. Questionnaire 3, administered three months after the visit, assessed satisfaction, discussed topics, and worries. The timing and content of the three questionnaires are summarized in Fig. 1. Before the visit, each participant received a personalized PfC based on their cancer diagnosis and treatment history, outlining individualized follow-up procedures and recommendations (Poplack et al., 2014). The consultation included medical history, physical examination, and referrals or additional tests if needed, and verbal and written information about individual long-term health risks.

### 2.3 Measures

#### 2.3.1 Satisfaction and worries

Satisfaction and worries were assessed using Questionnaire 3, which included the Princess Margaret Hospital Patient Satisfaction with Doctor Questionnaire (PMH/PSQ-MD; Loblaw et al., 1999). This 29-item questionnaire evaluates outpatient satisfaction in oncology settings and has been used in similar studies (Landen et al., 2003; Loblaw et al., 2004; Michel et al., 2011). For this study, the questionnaire was translated into German, culturally adapted to the Swiss context, and modified by removing the “does not apply” option. Two items were added to better capture worries after the consultation (“Since the consultation, I am more concerned about my health than before” and “Since the consultation at the clinic, my fears are greater than before”). Items were rated on a four-point Likert-scale (1 = exactly true to 4 = not true at all). Two ACCS representatives reviewed the German draft of the questionnaire, and their feedback was incorporated. Items were reverse-scored so that higher scores reflected greater satisfaction and worries. Two open-ended questions were excluded from the present analyses but reported elsewhere (Kull et al., 2025).

Principal component analysis of the adapted questionnaire yielded two satisfaction factors: patient-centered care (mean of 12 items; Cronbach's alpha = 0.73), and information dissemination (mean of nine items; Cronbach's alpha = 0.73). A third factor, worries (mean of three items; Cronbach's alpha = 0.81), was created. Details are provided in the Supporting Information Part A.

#### 2.3.2 Sociodemographic characteristics

Questionnaire 1 assessed gender, age at the LTFU visit, age at diagnosis, and migration background (defined as not holding Swiss citizenship, not being a Swiss citizen since birth, or not being born in Switzerland).

#### 2.3.3 Cancer-related characteristics

Cancer diagnoses, current health problems, and treatment intensity were derived from physicians' reports. Diagnoses were classified using ICCC-3. Time since diagnosis was calculated as age at study participation minus age at diagnosis. Treatment intensity was categorized according to Oeffinger et al. (2004): survivors receiving mantle or chest radiation, anthracyclines at cumulative doses ≥300 mg/m^2^, bleomycin, ifosfamide, or etoposide were classified as high risk; others as low risk.

General subjective vulnerability to late effects was assessed by asking ACCS to estimate the overall likelihood of experiencing any future late effects on a 0–100% scale.

#### 2.3.4 Consultation-related characteristics

In Questionnaire 2, participants rated whether they wished to discuss nine predefined topics (e.g., health status, health behavior, contraception; full list in Supporting Information Part C) on a five-point Likert scale (Loblaw et al., 2004). “Agree” or “very much agree” responses were classified as *wished*, “do not agree” or “do not agree at all” as *not wished*; neutral responses were excluded.

In Questionnaire 3, participants reported which topics they perceived as discussed during the consultation. Although all topics were addressed by the clinical team, survivors reported their subjective impression of which topics were discussed. A wish was considered fulfilled when a wished-for topic was reported as discussed, and the percentage of fulfilled wishes was calculated.

Motivation for attending LTFU was assessed using nine items rated from 1 = not at all important to 5 = very important. Based on previous research (Michel et al., 2016), two scales were created: *clinical care* (mean of five items; α = 0.76) and *supportive care* (mean of three items; α = 0.73).

#### 2.3.5 Psychosocial characteristics

Health-related quality of life (HRQoL) was assessed before the visit using the Short Form-36 version 2 (SF-36v2), which evaluates physical and mental HRQoL across eight health domains (Maruish, 2011; Morfeld et al., 2011; Ware and Sherbourne, 1992). Two summary scores were derived: the physical component score and the mental component score, with higher values indicating better HRQoL. Scores were based on Swiss normative data (T-scores; mean = 50, SD = 10) (Roser et al., 2019).

Processing of health information was measured using a modified Threatening Medical Situations Inventory (TMSI; Delisle et al., 2022), assessing *monitoring* (i.e. seeking information about potentially dangerous/painful medical situations) and *blunting* (i.e. distracting oneself from threatening situations or information). Participants rated six items (three per behavioral tendency) across four clinical scenarios on a five-point Likert-scale (1 = I would definitely not do that to 5 = I would definitely do that). Scores were calculated following Michel et al. (2011). A composite score (monitoring minus blunting) was calculated, and participants were dichotomized into *monitors* or *blunters* using a cut-off of 0.

### 2.4 Statistical analysis

Descriptive statistics were used to describe the sample (aim 1), with categorical variables presented as numbers and percentages and continuous variables as means and standard deviations. To identify factors associated with satisfaction and worries (aim 2), univariable and multivariable linear regression analyses were performed. Variables significant in univariable analyses (*p* < 0.05) were included in the multivariable models.

Because response times ranged from 0.40 to 37.70 months, participants responding more than 18 months after the visit were excluded from analyses related to discussion topics to reduce recall bias. All responses were included for satisfaction and worries, as general feedback was considered robust. Missing values in multi-item scales were imputed using the mean of completed items if at least 50% were available (Maruish, 2011). The proportion of imputed data was 6.41% for PMH/PSQ-MD, 1.44% for the motivation items, 2.63% for SF-36v2, and 0.92% for blunting and monitoring. Analyses were performed using Stata, Version 18.0 (StataCorp, 2023).

The study complied with the Declaration of Helsinki and was approved by the ethics committees of Bern (KEK BE) and Northwestern and Central Switzerland (EKNZ; 2017–00109). All participants provided informed consent.

## 3 Results

**Fig. 2:**
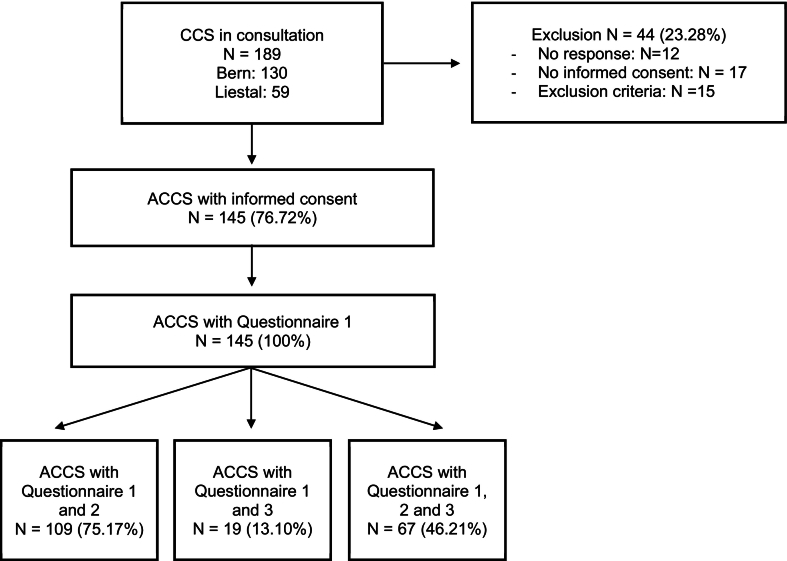
Flow chart of adult childhood cancer survivors included in the study of long-term follow-up clinic visits in Switzerland, 2017–2023. *Note*. CCS = Childhood Cancer Survivors, ACCS = Adult childhood cancer survivors. *Note*. CCS = Childhood Cancer Survivors, ACCS = Adult childhood cancer survivors.

Of the 189 ACCS, 145 (76.72%) provided informed consent and completed Questionnaire 1. Of these, 109 (75.17%) also completed Questionnaire 2 prior to the LTFU visit, and 67 (46.21%) completed Questionnaire 3 after the visit. Additionally, 19 participants (13.10%) returned only Questionnaires 1 and 3 (Fig. 2). All ACCS who completed at least one questionnaire were included in the analyses.

The characteristics of the study sample are shown in Table A. Around two-thirds of ACCS (68.53%; *N* = 98) received LTFU care in Bern. The sample included 60.69% (*N* = 88) female participants. Initial cancer diagnoses were diverse, reflecting the distribution of cancer types observed in children and adolescents. The most frequent diagnosis was leukemia (30.34%; *N* = 44). Mean age at diagnosis was 9.96 years (SD = 5.49; range: 0.20–19.90). Over half of the participants received high intensity therapy (59.31%; *N* = 86). Mean age at consultation was 31.54 years (SD = 11.42; range: 18–62). A minority of the study population had a migration background (15.94%; *N* = 22). On average, participants had 6.01 current health problems (SD = 3.70; range: 0–16), and their subjective expected likelihood of potential future late effects was rated at 35.57% (SD = 28.48; range: 0–100).

**Table A:** Descriptive characteristics of adult childhood cancer survivors attending long-term follow-up clinics in Switzerland, 2017–2023.

**Socio-demographic characteristics**				
		**N (%)**		
*Gender*				
	Male	57 (39.31)		
	Female	88 (60.69)		
*Migration background*^*a*^				
	No	116 (84.06)		
	Yes	22 (15.94)		

		**N**	**Mean (SD)**	**Range**
*Age at consultation (years)*		145	31.54 (11.42)	18.00-62.00
**Cancer-related characteristics**				
		**N (%)**		
*Cancer type (ICCC-3)*				
Leukemia		44 (30.34)		
Lymphoma		37 (25.52)		
Sarcoma		28 (19.31)		
Brain and other CNS Tumor		17 (11.72)		
Neuroblastoma		6 (4.14)		
Kidney tumor		5 (3.45)		
Other		3 (2.07)		
Liver tumor		2 (1.38)		
Germ cell tumor		1 (0.69)		
Nasopharyngeal carcinoma		1 (0.69)		
Retinoblastoma		1 (0.69)		
*Treatment*				
Low risk group		59 (40.69)		
High risk group		86 (59.31)		

		**N**	**Mean (SD)**	**Range**
*Age at diagnosis (years)*		145	9.96 (5.49)	0.20-19.90
*Time since diagnosis (years)*		145	21.58 (11.71)	2.80-53.80
*Number of current health problems*		144	6.01 (3.70)	0.00-16.00
*Subjective vulnerability for late effects (%)*		124	35.57 (28.48)	0.00-100.00
**Consultation-related characteristics**				
		**N (%)**		
*Consultation location*^a^				
Liestal		45 (31.47)		
Bern		98 (68.53)		

		**N**	**Mean (SD)**	**Range**
*Number of topics wished to be discussed*		69	6.59 (1.90)	2.00-9.00
*Proportion of wished topics discussed (%)*		64	79.15 (24.61)	0.00-100.00
*Motivation for follow-up: supportive care (rating 1-5)*		107	3.61 (0.85)	1.25-5.00
*Motivation for follow-up: clinical care (rating 1-5)*		107	4.34 (0.63)	2.25-5.00
*Satisfaction: Patient-centered care (rating 1-4)*		86	3.77 (0.28)	2.75-4.00
*Satisfaction: Information dissemination (rating 1-4)*		88	3.58 (0.33)	1.78-4.00
*Worries (rating 1-4)*		79	2.02 (0.71)	1.00-3.67

**Psychosocial characteristics**				
		**N (%)**		
*Processing of health information*				
Monitoring		62 (56.36)		
Blunting		48 (43.64)		
		**N**	**Mean (SD)**	**Range**
*Physical HRQoL (T-score)*		133	47.24 (11.22)	11.10- 63.43
*Mental HRQoL (T-score)*		133	47.63 (11.16)	7.81- 64.93

*Note*. Total sample size *N* = 145;; SD = standard deviation, ICCC-3 = International Classification of Childhood Cancer, Third edition, CNS = central nervous system, HRQoL = health-related quality of life.

a Missing values; percentages are based on available data.

Satisfaction was very high for both patient-centered care (mean = 3.77, SD = 0.28, range: 2.75–4.00; N = 86) and information dissemination (mean = 3.58, SD = 0.33, range: 1.78–4.00; N = 88). ACCS levels of worries after their LTFU visits were reported at moderate levels (mean = 2.02, SD = 0.71, range = 1.00–3.67, *N* = 79).

Results for the univariable regression analyses can be found in Table S1 (Supporting Information Part B). Multivariable regression models for satisfaction with patient-centered care and worries are shown in Table B, including all variables that were significant in the univariable analyses. No multivariable regression analysis was conducted for the third outcome, information dissemination, as no significant associations were found in the univariable analyses.

**Table B:** Multivariable linear regression analyses of factors associated with satisfaction with patient-centered care and worries among adult childhood cancer survivors attending long-term follow-up clinics in Switzerland, 2017–2023.

**Satisfaction: patient-centered care**		
	**Coeff**	**95% CI**
Treatment		
*Low intensity*	Ref	
*High intensity*	0.08	−0.05, 0.22
Consultation location		
*Liestal*	Ref	
*Bern*	0.18	0.05, 0.31
Proportion of wished topics discussed	0.00	0.00, 0.01
Mental HRQOL	0.00	−0.00, 0.01
**Worries**		
	**Coeff**	**95% CI**
Age at consultation	−0.00	−0.04, 0.04
Time since diagnosis	0.01	−0.03, 0.05
Number of current health problems	0.01	−0.06, 0.07
Subjective vulnerability	0.00	−0.01, 0.01
Motivation: supportive care	0.03	−0.23, 0.30
Physical HRQOL	−0.01	−0.03, 0.01
Mental HRQOL	−0.02	−0.04, −0.00
Processing of health information		
*Monitoring*	Ref	
*Blunting*	−0.22	−0.68, 0.23

*Note*. HRQoL = health-related quality of life; Coeff = coefficient; CI = confidence interval; Ref = reference category. Only variables significantly associated with satisfaction or worries in univariable regression analysis were included.

The overall multivariable regression model for satisfaction with patient-centered care was significant, F(4, 48) = 6.11, *p* < 0.01, and explained 33.80% of the variance (R^2^ = 0.34). Receiving the consultation in Bern (compared to Liestal) was associated with higher satisfaction (B = 0.18, *p* = 0.01). In addition, a higher percentage of wished topics discussed during the LTFU visit was positively associated with satisfaction (B = 0.00, *p* = 0.01;).

The overall multivariable regression model for worries was also significant, F(8, 43) = 2.46, *p* = 0.03, and explained 31.40% of the variance (*R*^*2*^ = 0.31;). Lower mental HRQoL was significantly associated with increased worries (B = -0.02, *p* = 0.03).

## 4 Discussion

This study examined satisfaction and worries among ACCS attending LTFU consultations at two specialized clinics. Overall satisfaction was very high, and a higher proportion of wished topics addressed during the consultation was associated with greater satisfaction. However, ACCS reported moderate levels of worries after the visit, particularly those with lower baseline mental HRQoL.

The high satisfaction observed is consistent with previous research on follow-up consultations (Delisle et al., 2022; Hill et al., 2023; Tinner et al., 2024). By additionally assessing worries after the consultation, our findings highlight that some survivors may experience increased concerns despite high satisfaction. This underlines the importance of addressing psychosocial issues during LTFU visits, as cancer survivors often expect psychological and emotional support as part of follow-up care (Brandenbarg et al., 2017). Fear of cancer recurrence has been identified as a barrier to follow-up attendance (Smits-Seemann et al., 2017), although this could not be examined in our study because worries were assessed only after participants had attended the LTFU visit.

Satisfaction with patient-centered care differed between sites (Liestal vs. Bern) whereas a review of breast-cancer follow-up found that the location of care did not affect survivors' positive attitudes toward follow-up (Collins et al., 2004). The differing findings likely reflect differences in recruitment pathways and expectations. While most ACCS in Bern transitioned directly from pediatric care or a related cardio-oncology study, ACCS in Liestal often self-referred and traveled longer distances, possibly resulting in higher expectations that could not always be met during a single visit. This discrepancy may have contributed to the lower satisfaction ratings observed in Liestal, as discussed in previous research (Cleary et al., 2017).

Importantly, a higher proportion of wished topics being addressed during the consultation was significantly associated with greater satisfaction. While previous studies reported higher satisfaction with more topics discussed (Absolom et al., 2006; Michel et al., 2011), our findings suggest that the alignment between survivors' individual expectations and topics discussed may be particularly relevant. Supporting this, a study from China using a random sample of hospital patients identified patients' expectations as the most influential psychosocial factor affecting satisfaction with care (Wang et al., 2023).

No significant predictors emerged for satisfaction with information dissemination. In contrast, a previous study from Sheffield found that satisfaction with information exchange was higher among ACCS who had longer consultations, discussed more topics, and reported higher mental HRQoL (Michel et al., 2011). As information provision was individualized and delivered by the same provider in our study, variability in perceived information quality may have been limited.

No sociodemographic or cancer-related factors were associated with satisfaction or worries, consistent with a Canadian study of adult-onset cancer survivors (Delisle et al., 2022). In that study, good or very good quality of life and emotional health were important predictors of satisfaction. While HRQoL was not associated with satisfaction in our study, lower mental HRQoL was significantly associated with higher levels of worries following the consultation, suggesting it may represent a risk factor for increased worries. This association may reflect underlying anxiety or depressive symptoms.

The primary aim of LTFU care is the early identification of cancer- and cancer-treatment-related late effects and health problems (Skinner et al., 2007), which requires survivors to attend LTFU visits. Patient satisfaction plays a crucial role in fostering such engagement, as does minimizing the emergence of worries related to LTFU care. In our study, survivors reported moderate levels of worry after LTFU visits, suggesting that integrating psychosocial counseling into routine care and explicitly addressing concerns during consultations may help alleviate these worries. ACCS who are at risk of elevated worry levels - particularly those with lower mental HRQoL - may benefit from targeted psychological support and additional resources.

To improve satisfaction, structured pre-visit questionnaires could help align consultations with survivors' preferences and concerns. However, limited reimbursement within the Swiss healthcare system may restrict the feasibility of additional preparatory work.

A key strength of this study is the diverse survivor population, reflecting the heterogeneity of the broader childhood cancer population, and the availability of comprehensive cancer-related information, including detailed treatment data. The two study centers are different in size and specialization, demonstrating that interdisciplinary survivorship care is feasible in different settings and supporting the general applicability of this approach. However, several limitations should be considered. First, the relatively small sample size and attrition in completing the post-visit questionnaire may have limited statistical power and reduced the ability to detect subtle effects. Second, ACCS were not actively recruited but either self-referred or were referred to by healthcare professionals, which may have introduced selection bias. Differences in recruitment pathways between the two sites may also have affected satisfaction ratings. In addition, satisfaction with the first consultation at the participating adult LTFU clinic may have been influenced by survivors' previous experiences in pediatric oncology or other follow-up settings, as well as by expectations formed before entering the program. Third, the questionnaires were long and potentially burdensome, particularly for ACCS experiencing cognitive late effects, possibly further amplifying selection bias by excluding individuals less able to complete the assessments. Fourth, the inclusion of only two centers within one healthcare system and country limits generalizability of the findings to other settings or healthcare contexts and may introduce center-specific biases. Finally, responses accepted up to 18 months after the visit may have been affected by recall bias.

## 5 Conclusions

Although overall satisfaction with the LTFU visit was very high, some ACCS reported worries after their LTFU consultation, particularly those with lower mental HRQoL. These individuals may benefit from targeted psychological support. Satisfaction was not associated with sociodemographic or cancer-related characteristics but was related to whether ACCS felt their individual preferences and needs were taken into account during LTFU care. Future research should further examine how worries develop after LTFU visits and whether tailored interventions can reduce distress in vulnerable subgroups. Clinically, the findings highlight the importance of systematically addressing psychological well-being during LTFU visits and tailoring care to individual needs and preferences. These results are particularly relevant given that, in recent years, similar long-term follow-up structures have been established outside Switzerland, suggesting that our findings may be transferable to comparable healthcare contexts.

## CRediT authorship contribution statement

**Sara Buchmüller:** Writing – review & editing, Writing – original draft, Visualization, Validation, Methodology, Formal analysis, Data curation. **Sonja Kälin:** Writing – review & editing, Writing – original draft, Visualization, Validation, Methodology, Investigation, Formal analysis, Conceptualization. **Martina Ospelt:** Writing – review & editing, Methodology, Formal analysis, Conceptualization. **Gisela Michel:** Writing – review & editing, Methodology, Conceptualization. **Carole E. Aubert:** Writing – review & editing, Methodology, Conceptualization. **Jörg D. Leuppi:** Writing – review & editing, Methodology, Funding acquisition, Conceptualization. **Maria M. Wertli:** Writing – review & editing, Validation, Supervision, Resources, Project administration, Funding acquisition, Formal analysis, Data curation, Conceptualization. **Eva Maria E. Tinner:** Writing – review & editing, Writing – original draft, Visualization, Validation, Supervision, Project administration, Methodology, Investigation, Funding acquisition, Formal analysis, Data curation, Conceptualization.

## Ethical approval

The study was conducted in accordance with the Declaration of Helsinki, and approved by ethics committee Bern (KEK BE) under the lead of the ethical committee of northwestern and central Switzerland (EKNZ) with the number 2017–00109. Participants gave informed consent to participate in the study before taking part.

## Funding

This work was supported by the science funds of the University Institute of Internal Medicine of the cantonal hospital Baselland (KSBL) (grant number: N/A), the Basel Region Childhood Cancer Foundation (grant number: #2020-F009) and the “Berner Stiftung für krebskranke Kinder und Jugendliche” (grant number: N/A). CEA was funded by the Swiss National Science Foundation (Ambizione Grant PZ00P3_201672). SK and MO were funded by the Swiss Cancer Research Foundation (Grant no. KFS-5384-08-2021).

## Declaration of competing interest

The authors declare that they have no known competing financial interests or personal relationships that could have appeared to influence the work reported in this paper.

## Data Availability

Data are available from the corresponding author upon reasonable request.
