# Supplementary material for: Satisfaction with follow-up consultations among Swiss childhood cancer survivors: A prospective cohort study
Source: Prev Med Rep. 2026 Jul 27;69:103591. doi: 10.1016/j.pmedr.2026.103591 (PMC13450578; doi:10.1016/j.pmedr.2026.103591)
Supplement: Supplementary file 1 [file mmc1.docx]

**Supporting Information File**

**Satisfaction with follow-up consultations among Swiss childhood cancer survivors: a prospective cohort study**

Sara Buchmüller^1^§, Sonja Kälin^2^§, Martina Ospelt^2^, Gisela Michel^2^, Carole E. Aubert^1,4^, Jörg D. Leuppi^3^, Maria M. Wertli^1, 5^#, Eva Maria E. Tinner^3,6^ #,

§Shared first authorship: equal contribution

#Shared last authorship: equal contribution

**Affiliations:**

1. Department of General Internal Medicine, Inselspital, Bern University Hospital, University of Bern, Bern, Switzerland
2. Faculty of Health Sciences and Medicine, University of Lucerne, Lucerne, Switzerland
3. University Institute of Internal Medicine, Cantonal Hospital Baselland, Liestal, Switzerland; and Medical Faculty University of Basel, Basel, Switzerland
4. Institute for Primary Healthcare (BIHAM), University of Bern, Bern, Switzerland
5. Department of Internal Medicine, Cantonal Hospital Baden, Baden, Switzerland
6. Division of Hematology/Oncology, Department of Pediatrics, Inselspital Bern, University of Bern, Bern, Switzerland

**Overview:**

**Supporting Information A –** Principal component analysis to extract the satisfaction and worries scales

**Supporting Information B –** Table S1: Univariable linear regression analysis with the different domains of satisfaction as dependent variables.

**Supporting Information C –** List of topics wished to be discussed

**Supporting Information A - Principal component analysis to extract the satisfaction and worries scales**

Principal component analysis (PCA) with orthogonal varimax rotation was conducted to determine the questionnaire scales. As there were a number of complicated double negation items in the questionnaire, a preliminary analysis using item-rest-correlations was carried out to identify items with very low correlations (<0.10) with the other items, resulting in the exclusion of two items from further analysis. One item ('The doctor was NOT friendly to me') had to be excluded due to zero variance, as all participants rated it as 4 ('strongly disagree'). Scree test and parallel analysis both indicated the extraction of two factors. The Bartlett's test statistic was 577, yielding a p-value of <0.01. Four additional items were excluded due to low loadings on either factor. The single item assessing worries (‘After my visit to the doctor, I feel much better about my concerns’) was also excluded from the PCA, as it was part of a separately defined factor (‘worries’; see below).

The first identified factor, comprising 12 items, was labelled 'patient-centered care' and described the satisfaction in emotional and non-verbal skills of the clinician as well as the time management. The second identified factor, comprising 9 items, was labelled 'information dissemination' and described satisfaction with the content provided during the visit, including whether the information was understood and deemed adequate, as well as the perceived honesty of the clinician. Cronbach's alpha was sufficient for both factors, with factor one 'patient-centered care' at 0.73 and factor two 'information dissemination' at 0.70.

An additional factor for 'worries' was created, consisting of three items. One item was from the original questionnaire ('After my visit to the doctor, I feel much better about my concerns') and two items were created by the authors. This factor, labelled 'worries', showed very good internal consistency (Cronbach's alpha = 0.81).

**Supporting Information B**

TABLE S1: Univariable linear regression analyses of factors associated with satisfaction domains among adult childhood cancer survivors attending long-term follow-up clinics in Switzerland, 2017–2023.

|  | **Patient-Centered Care** | |  | **Information Dissemination** | |  | **Worries** | |
| --- | --- | --- | --- | --- | --- | --- | --- | --- |
| **Predictors** | **Coeff** | **p** |  | **Coeff** | **p** |  | **Coeff** | **p** |
| Gender |  | 0.49 |  |  | 0.78 |  |  | 0.18 |
| *male* | Ref |  |  | Ref |  |  | Ref |  |
| *female* | 0.54 |  |  | 0.19 |  |  | 0.70 |  |
| Age at consultation | -0.05 | 0.17 |  | 0.03 | 0.27 |  | 0.05 | 0.02 |
| Migration background | | 0.43 |  |  | 0.80 |  |  | 0.78 |
| *no* | Ref |  |  | Ref |  |  | Ref |  |
| *yes* | -0.82 |  |  | 0.23 |  |  | 0.18 |  |
| Age at diagnosis | 0.04 | 0.56 |  | 0.01 | 0.90 |  | 0.02 | 0.72 |
| Time since diagnosis | -0.06 | 0.09 |  | 0.03 | 0.28 |  | 0.05 | 0.03 |
| Number of current health problems | -0.08 | 0.45 |  | -0.02 | 0.83 |  | 0.16 | 0.02 |
| Subjective vulnerability | -0.02 | 0.27 |  | -0.01 | 0.31 |  | 0.03 | <0.01 |
| Treatment |  | 0.02 |  |  | 0.78 |  |  | 0.54 |
| *Low intensity* | Ref |  |  | Ref |  |  | Ref |  |
| *High intensity* | 1.72 |  |  | 0.18 |  |  | -0.31 |  |
| Consultation location | | 0.02 |  |  | 0.82 |  |  | 0.07 |
| *Liestal* | Ref |  |  | Ref |  |  | Ref |  |
| *Bern* | 1.76 |  |  | 0.15 |  |  | -0.87 |  |
| Length of consultation | -0.08 | 0.76 |  | -0.28 | 0.20 |  | -0.03 | 0.86 |
| Number of wished topics | 0.24 | 0.26 |  | 0.21 | 0.16 |  | -0.06 | 0.65 |
| Proportion of wished topics discussed | 0.06 | <0.01 |  | 0.02 | 0.40 |  | -0.01 | 0.35 |
| Motivation: supportive care | -0.79 | 0.09 |  | 0.18 | 0.69 |  | 0.77 | 0.02 |
| Motivation: clinical care | -0.09 | 0.89 |  | 0.26 | 0.69 |  | 0.08 | 0.87 |
| Physical HRQOL | 0.02 | 0.55 |  | -0.01 | 0.71 |  | -0.06 | <0.01 |
| Mental HRQOL | 0.09 | <0.01 |  | -0.02 | 0.60 |  | -0.07 | <0.01 |
| Processing of health information |  | 0.23 |  |  | 0.69 |  |  | 0.01 |
| *Monitoring* | Ref |  |  | Ref |  |  | Ref |  |
| *Blunting* | 0.95 |  |  | -0.30 |  |  | -1.44 |  |

*Note*. HRQOL = health-related quality of life; Coeff = coefficient; Ref = reference category

**Supporting Information C – List of topics wished to be discussed**

Which topics would you like to discuss with the staff of the interdisciplinary follow-up clinic at your next appointment?

Topics:

1. My current state of health
2. Medication
3. Long-term effects of the oncological therapy or the illness
4. My current health behavior (e.g., smoking, alcohol consumption, exercise, nutrition)
5. Contraception
6. Fertility
7. Sexual problems
8. Problems at work / in education
9. Insurance (e.g., life insurance)
10. Can you think of any other reasons? If yes, please list them below
